# Assessment of Epidemiology Capacity in State Health Departments — United States, 2021

**DOI:** 10.15585/mmwr.mm7113a2

**Published:** 2022-04-01

**Authors:** Jessica Arrazola, Sarah Auer

**Affiliations:** 1 Council of State and Territorial Epidemiologists, Atlanta, Georgia.

In 2021, during the COVID-19 response, the Council of State and Territorial Epidemiologists (CSTE) conducted its seventh periodic Epidemiology Capacity Assessment (ECA), a national assessment that evaluates trends in applied epidemiology workforce size, funding, and epidemiology capacity at state health departments.* A standardized web-based questionnaire was sent to state epidemiologists in 50 states and the District of Columbia (DC). The questionnaire assessed the number of current and optimal epidemiologist positions; sources of epidemiology activity and personnel funding; and each health department’s self-perceived capacity to lead activities, provide subject matter expertise, and obtain and manage resources for the three essential public health services (EPHS) most closely linked to epidemiology.^†^ CSTE enumerated 4,136 epidemiology positions across the United States, with an additional 2,196 positions needed to provide basic public health services. From 2017 to 2021, the number of epidemiologists in state health departments increased 23%, an increase primarily accounted for by the number of those supporting the COVID-19 response^§^. The number of staff members decreased in program areas of infectious diseases, chronic diseases, and maternal and child health (MCH). Federal funding supports most epidemiology activities (85%) and epidemiology personnel (83%). Overall capacity to deliver the EPHS has declined, and epidemiology workforce and capacity needs remain unmet. More epidemiologists and sustainable funding are needed to consistently and effectively deliver EPHS. Additional resources (e.g., funding for competitive compensation and pathways for career advancement) are essential for recruitment and retention of epidemiologists to support public health activities across all program areas.

The ECA questionnaire instrument was updated in 2021 to include new epidemiology program areas for generalists and COVID-19 specialists and the revised EPHS (*1*). The COVID-19 program area sought to capture epidemiologists who were added for the COVID-19 response or reallocated for response efforts, separate from general infectious disease capacity. A set of core questions has remained essentially unchanged and permits monitoring of trends in the epidemiology workforce employed by the 50 states, DC, U.S. territories, and freely associated states, including current funding sources for epidemiology activities and personnel, capacity in the three EPHS relevant to epidemiology, and issues faced by health departments in recruitment, retention, and training of skilled epidemiologists to meet current needs and evolving priorities.

After CSTE pilot-tested the questionnaire instrument, the 2021 ECA was disseminated electronically to the lead state and territorial epidemiologist for each jurisdiction, using Qualtrics,^¶^ an online survey tool. Data collection began January 11, 2021, and was completed April 1, 2021. Virtual technical assistance was provided to support the completion of the ECA. All 50 states, DC, and four territories responded to the assessment; this analysis includes responses only from U.S. states and DC. The number of full-time equivalent (FTE) epidemiologist positions (rounded to the nearest 0.1 FTE) by program area and source of funding was collected. For purposes of the ECA, CSTE defined capacity as “the state health department’s ability to lead activities, provide subject matter expertise, and apply for, receive, and manage resources to conduct key activities.” Respondents subjectively evaluated their capacity** as none (0%), minimal (1%–24%), partial (25%–49%), substantial (50%–74%), almost full (75%–99%), and full (100%). Data were analyzed using SAS (version 9.4; SAS Institute). This activity was reviewed by CDC and was conducted consistent with applicable federal law and CDC policy.^††^

Respondents from 50 states and DC reported that 4,136 FTE epidemiologists were working in state health departments in 2021, a 23% increase over the 3,370 reported in 2017 (*2*). Overall, the number of epidemiologists per 100,000 population was 1.26 (range = 0.13–7.58), 21% higher than the 1.04 per 100,000 calculated in 2017. The size of the epidemiology workforce in each jurisdiction ranged from four to 255 FTEs.

Epidemiology activities in 2021 were supported in large part by federal funds (85%, an increase of 8% from 2017), followed by state funds (12%) and other sources (3%). As part of the federal funding for epidemiology activities, 39% was designated for COVID-19 activities with time-limited funding. The federal government funds 85% of epidemiology personnel positions, with 33% of these funds designated specifically for COVID-19 personnel. The remaining epidemiology personnel positions are funded by state government (15%) and other sources of funding (2%). Federal funding supports approximately 80% of epidemiology positions for COVID-19 response, preparedness, and substance use. In contrast, state and other sources of funding support approximately 50% of informatics, environmental health, generalist, and vital statistics positions.

Among program areas, infectious disease accounted for 1,498 (36%) of the 4,136 epidemiology positions, followed by COVID-19 response (24%) and MCH (7%) (Table 1). Program areas with the fewest epidemiologists included genomics, mental health, oral health, and occupational health. Most of the overall increase in workforce size can be attributed to new positions supporting the COVID-19 response.

**TABLE 1:** Full-time equivalent epidemiologist positions, by program area — Council of State and Territorial Epidemiologists Epidemiology Capacity Assessment, United States, 2021

Program area	Current no. (%)	Additional positions needed	Optimal no.* (%)^†^	Vacant positions^§^	Positions actively recruiting, no. (% of vacant positions)^¶^
Infectious diseases	1,498 (36.2)	562	2,059 (72.7)	182	137 (75.2)
COVID-19 response	978 (23.7)	454	1,432 (68.3)	362	304 (83.9)
Maternal and child health	292 (7.1)	135	428 (68.3)	40	27 (67.5)
Chronic disease	250 (6.0)	153	402 (62.1)	40	30 (75.0)
Environmental health	231 (5.6)	135	366 (63.2)	18	13 (72.2)
Informatics	198 (4.8)	166	364 (54.4)	45	37 (82.2)
Preparedness	128 (3.1)	74	201 (63.4)	10	10 (100.0)
Injury	126 (3.0)	66	192 (65.8)	10	8 (80.0)
Vital statistics	117 (2.8)	62	179 (65.2)	13	10 (76.9)
Substance use	114 (2.8)	64	178 (64.2)	14	8 (57.1)
Generalist	81 (2.0)	85	166 (49.1)	7	3 (42.8)
Other	55 (1.3)	60	115 (48.1)	102	93 (91.1)
Occupational health	34 (0.8)	48	82 (41.2)	2	1 (50.0)
Oral health	20 (0.5)	31	52 (39.2)	2	0 (NA)
Mental health	9 (0.2)	57	66 (13.2)	2	2 (100.0)
Genomics**	5 (0.1)	46	51 (9.8)	3	5 (NA)
**Total**	**4,136**	**2,196**	**6,333 (65.3)**	**852**	**688 (80.8)**

**Abbreviation:** NA = not applicable.

* Positions currently filled plus additional positions needed.

^†^ Positions currently filled as a percentage of the ideal number of positions.

^§^ Positions to be filled at a state health department for which work was available and the job could begin within 30 days.

^¶^ Vacant positions that human resource organizations were actively working to fill.

** The difference in the number of vacant positions and positions actively being recruited for this program area is likely because of new positions that are going to be created versus existing vacancies.

The largest absolute and relative increases between 2017 and 2021 were in informatics, where 103 positions were added, representing a 107% increase (Table 2). Since 2017, infectious diseases positions decreased 19% (loss of 341 epidemiologists), chronic diseases decreased 18% (loss of 55 epidemiologists), and MCH decreased 9% (loss of 29 epidemiologists).

**TABLE 2:** Full-time equivalent epidemiologist positions and absolute and percent change, by program area during 2017 and 2021 — Council of State and Territorial Epidemiologists Epidemiology Capacity Assessment, United States, 2021

Program area	2017	2021	Change, no. (%)
Other*	143.4	55.2	−88.2 (−61.5)
Infectious diseases	1,838.2	1,497.7	−340.5 (−18.5)
Chronic disease	304.4	249.9	−54.5 (−17.9)
Maternal and child health	321.2	292.2	−29.0 (−9.0)
Environmental health	221.7	231.4	9.6 (4.3)
Vital statistics	110.7	116.7	6.0 (5.4)
Preparedness	117.6	127.5	9.9 (8.4)
Oral health	18.0	20.2	2.2 (12.2)
Genomics	4.4	5.0	0.6 (13.6)
Occupational health	28.4	33.8	5.4 (19.0)
Injury	102.5	126.1	23.6 (22.9)
Substance use	58.6	114.0	55.4 (94.6)
Informatics	95.7	198.4	102.7 (107.3)
Mental health	4.0	8.7	4.7 (117.5)
COVID-19 response^†^	NA	977.5	NA
Generalist^†^	NA	81.4	NA

**Abbreviations:** FTE = full-time equivalent; NA = not applicable.

* The other program area included FTEs from program areas including, but not limited to, health equity, community health, health disparities, refugee health, and minority health.

^†^ COVID-19 response and generalist program areas were added to the 2021 Epidemiology Capacity Assessment to capture data for epidemiologists working on the COVID-19 response and epidemiologists working across a variety of program areas.

Participating state epidemiologists expressed the need for an additional 2,196 epidemiologists to deliver the EPHS, a 53% increase over the current number (Table 1). The largest number of positions needed were in infectious diseases (562), COVID-19 response (454), informatics (166), chronic diseases (153), MCH (135), and environmental health (135). The largest proportional increases needed were in genomics (922% increase, from five to 51), mental health (656% increase, from nine to 66), oral health (155% increase, from 20 to 52), and occupational health (143% increase, from 34 to 82). At the time of the assessment, among 852 position vacancies nationwide, 688 (81%) were being actively recruited. Filling these vacancies will address only 31% of the estimated additional 2,196 positions needed.

In 2021, 75% of jurisdictions had substantial-to-full capacity for monitoring health status (EPHS 1) and 88% capacity for diagnosing and investigating health problems and hazards (EPHS 2); both represented declines from 2017 (84% and 92%, respectively). Substantial-to-full capacity to conduct research and evaluation (EPHS 9) was 43%.^§§^

When overall capacity was examined by program area, substantial-to-full capacity was highest for infectious diseases (88%), MCH (70%), chronic diseases (66%), vital statistics (54%), substance use (52%), injury (50%), and preparedness (50%) (Figure). States reported minimal-to-no capacity in genomics (90%) and mental health (78%). Since 2017, there was a decline in the proportion of states reporting substantial-to-full capacity in preparedness (17%), chronic disease (12%), and infectious disease (8%). In contrast, there was an increase in the proportion of states reporting substantial-to-full capacity in the areas of substance use (36%), informatics (17%), mental health (12%), occupational health (10%), and oral health (10%).

**FIGURE:**
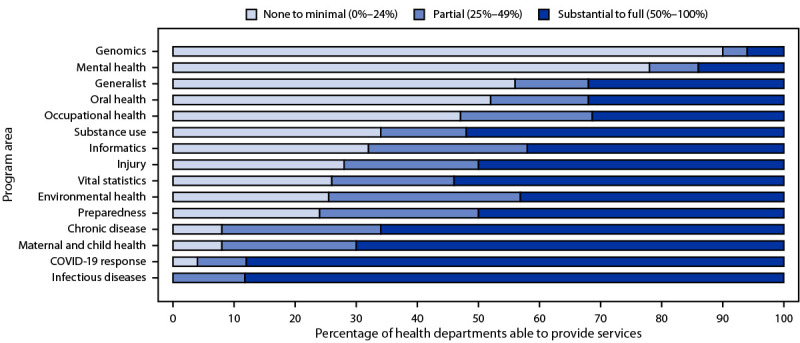
Overall epidemiologic capacity to provide essential public health services* — Council of State and Territorial Epidemiologists Epidemiology Capacity Assessment, United States, 2021 **Abbreviations:** ECA = Epidemiology Capacity Assessment; EPHS = essential public health service. * The 2021 ECA measured EPHS 1 (assess and monitor population health status, factors that influence health, and community needs and assets), EPHS 2 (investigate, diagnose, and address health problems and hazards affecting the population), and EPHS 9 (improve and innovate public health functions through ongoing evaluation, research, and continuous quality improvement).

## Discussion

Despite achieving the largest applied epidemiology workforce since tracking began in 2001, reported decreases in current staffing levels and an increased need for staff members by state health departments are concerning. Decreases in the number of staff members have important impacts on the ability of public health agencies to detect, investigate, and respond (*3*) to a myriad of critical threats, including infectious and chronic diseases and environmental hazards. Without accurate information about these conditions and the populations they affect, public health agencies cannot take appropriate actions to reduce or prevent illnesses, long-term sequelae, and death. The decline in the number of existing workers to support areas outside of COVID-19 is further compounded by the need for more skilled epidemiologists across all program areas. Limited staffing adversely affects public health workforce morale, mental health (*4*), and the ability to engage with and support non–COVID-19 priorities.

Despite the influx of COVID-19 epidemiologists, COVID-19 funding is short-term and unable to support staffing and programmatic capacity beyond 2026 (*5*). Across the country, COVID-19 funding supports an average of 33% (range = 0%–94%) of epidemiology personnel, leaving these positions vulnerable without sustainable funding. Jurisdictions need to develop strategies to integrate the temporary COVID-19 workforce into long-term positions and to invest in core capacity to address future emergencies and public health threats. Staffing strategies should consider positions with specialized and diverse skill sets, including epidemiologists, data scientists, laboratorians, and informaticians to support the data and systems infrastructure. Technologies such as genomic sequencing and electronic laboratory reports should be leveraged to support the development and use of data infrastructure supporting epidemiological investigation and response. Epidemiology leaders can demonstrate the value and utility of epidemiologists and epidemiology services across programs and the broader public health department. The growth of epidemiology infrastructure requires the integration of epidemiologists across programs and their budgets; provision of opportunities to learn and apply new skills among existing staff members, especially development of epidemiology leaders; creation of expedited hiring career pathways to retain temporary staff members, and incorporation of standard epidemiology job classifications and career ladders, such as those based on the Applied Epidemiology Competencies.^¶¶^

The findings in this report are subject to at least two limitations. First, the number of epidemiology positions is measured only for state health departments and does not include epidemiologists working in other state agencies (e.g., occupational health epidemiologists working in state departments of labor). Second, data on public health capacity are subjective; the data reflect the jurisdiction’s needs at the time of fielding, which might be biased toward immediate priorities, such as the COVID-19 response, rather than toward routine public health activities and planned strategic priorities or the resources to support public health transformation.

The Coronavirus Aid, Relief, and Economic Security Act has authorized $500 million for public health data surveillance and analytics infrastructure modernization.*** The American Rescue Plan Act of 2021 authorized $7.66 billion for public health response activities including, but not limited to, workforce recruitment, hiring, retention, and training.^†††^ These are essential investments to bolster public health infrastructure; however, this cannot be accomplished without long-term sustainable support that does not rely on temporary emergency public health funding. Transforming the public health infrastructure by harnessing the power of technology and building a permanent workforce capable to deliver EPHS in a post–COVID-19 era is critical.

**Summary**

**What is already known about this topic?**

The COVID-19 response has strained the U.S. public health system. Although the state health department epidemiology workforce has increased since 2017, workforce and capacity needs remain unmet.

**What is added by this report?**

From 2017 to 2021, the number of epidemiologists in state health departments increased 23%, primarily because of those supporting the COVID-19 response. The epidemiology workforce remains substantially understaffed, and core program areas have experienced staffing declines. Temporary federal funding has increased to support 85% of epidemiology activities and 83% of personnel. Overall capacity to deliver essential public health services has declined.

**What are the implications for public health practice?**

More epidemiologists and sustainable resources are needed to deliver essential public health services consistently and effectively.

## Footnotes

* ECAs were conducted in 2001, 2004, 2006, 2009, 2013, and 2017, with supplementary workforce enumeration conducted in 2010. Since 2004, 100% of states and DC have responded to the assessment. When referring to state health departments, the ECA includes data from DC.

† The three EPHS capacities evaluated in the ECA were 1) assess and monitor population health status, factors that influence health, and community needs and assets (EPHS 1); 2) investigate, diagnose, and address health problems and hazards affecting the population (EPHS 2); and 3) improve and innovate public health functions through ongoing evaluation, research, and continuous quality improvement (EPHS 9).

§ Epidemiologists were defined in the ECA as “all those employed by the state; all those working at the state level who are either federal assignees (e.g., [Epidemic Intelligence Services officer], [Career Epidemiology Field officer], [Public Health Associate Program associate]) or contract employees (e.g., [Council of State and Territorial Epidemiologists] trainee, contracted from school of public health to work at or for the State Health Department); and state employees assigned to work at a local or regional level (e.g., to conduct investigations for a region of the state)” who should focus on the functions performed by the individual person rather than the job title, using as guidance the Applied Epidemiology Competencies.

¶
https://www.qualtrics.com/

** Capacity was defined in the ECA as “the state health department’s ability to lead activities, provide subject matter expertise, and apply for, receive, and manage resources to conduct key activities.”

†† 45 C.F.R. part 46, 21 C.F.R. part 56; 42 U.S.C. Sect. 241(d); 5 U.S.C. Sect. 552a; 44 U.S.C. Sect. 3501 et seq.

§§ In 2017, 39% of states and DC had substantial capacity in EPHS 9, and 43% had substantial capacity in 2021; however, EPHS 9 now measures both research and evaluation, unlike in 2017 when these program areas were measured separately.

¶¶
https://www.cste.org/group/CSTECDCAEC

***
https://www.congress.gov/bill/116th-congress/house-bill/748/text

†††
https://www.congress.gov/bill/117th-congress/house-bill/1319/text#toc-H1858361F2D3F4367B9C0A3F11D488654
